# Using Plasma Viral Load to Guide Antiretroviral Therapy Initiation to Prevent HIV-1 Transmission

**DOI:** 10.1371/journal.pone.0051192

**Published:** 2012-11-30

**Authors:** Pamela M. Murnane, James P. Hughes, Connie Celum, Jairam R. Lingappa, Nelly Mugo, Carey Farquhar, James Kiarie, Anna Wald, Jared M. Baeten, for the Partners in Prevention HSV/HIV Transmission Study Team

**Affiliations:** 1 Department of Epidemiology, University of Washington, Seattle, Washington, United States of America; 2 Department of Global Health, University of Washington, Seattle, Washington, United States of America; 3 Department of Biostatistics, University of Washington, Seattle, Washington, United States of America; 4 Department of Medicine, University of Washington, Seattle, Washington, United States of America; 5 Department of Pediatrics, University of Washington, Seattle, Washington, United States of America; 6 Department of Obstetrics & Gynaecology, University of Nairobi, Nairobi, Kenya; 7 Department of Obstetrics & Gynaecology, Kenyatta National Hospital, Nairobi, Kenya; 8 Department of Laboratory Medicine, University of Washington, Seattle, Washington, United States of America; 9 Vaccine and Infectious Disease Division, Fred Hutchinson Cancer Research Center (FHCRC), Seattle, Washington, United States of America; 10 Public Health Sciences and Clinical Research Divisions FHCRC, Seattle, Washington, United States of America; Institut Pasteur of Shanghai, Chinese Academy of Sciences, China

## Abstract

**Background:**

Current WHO guidelines recommend antiretroviral therapy (ART) initiation at CD4 counts ≤350 cells/µL. Increasing this threshold has been proposed, with a primary goal of reducing HIV-1 infectiousness. Because the quantity of HIV-1 in plasma is the primary predictor of HIV-1 transmission, consideration of plasma viral load in ART initiation guidelines is warranted.

**Methods:**

Using per-sex-act infectivity estimates and cross-sectional sexual behavior data from 2,484 HIV-1 infected persons with CD4 counts >350 enrolled in a study of African heterosexual HIV-1 serodiscordant couples, we calculated the number of transmissions expected and the number potentially averted under selected scenarios for ART initiation: i) CD4 count <500 cells/µL, ii) viral load ≥10,000 or ≥50,000 copies/mL and iii) universal treatment. For each scenario, we estimated the proportion of expected infections that could be averted, the proportion of infected persons initiating treatment, and the ratio of these proportions.

**Results:**

Initiating treatment at viral load ≥50,000 copies/mL would require treating 19.8% of infected persons with CD4 counts >350 while averting 40.5% of expected transmissions (ratio 2.0); treating at viral load ≥10,0000 copies/mL had a ratio of 1.5. In contrast, initiation at CD4 count <500 would require treating 41.8%, while averting 48.4% (ratio 1.1).

**Conclusion:**

Inclusion of viral load in ART initiation guidelines could permit targeting ART resources to HIV-1 infected persons who have a higher risk of transmitting HIV-1. Further work is needed to estimate costs and feasibility.

## Introduction

Antiretroviral therapy (ART) for treatment of HIV-1 infection has significant prevention benefits, reducing the risk of secondary HIV-1 transmission by over 90% [1], [2]. Mathematical models suggest that providing ART to all HIV-1 infected persons and achieving sustained viral suppression could substantially reduce population-level HIV-1 incidence [3], [4]. Earlier treatment is now recommended in many settings as a result of mounting evidence for treatment and prevention benefits of ART initiation early in the course of HIV-1 infection [5], [6]. Guidelines for initiation of ART for HIV-1 infected persons have generally been based on CD4 T cell count and symptoms of advanced HIV-1 disease: current WHO guidelines recommend ART initiation for WHO clinical stage 3 and 4 infections and at CD4 counts ≤350 cells/µL regardless of stage [7]; the European AIDS Clinical Society recommends ART start at any CD4 count for symptomatic disease and consideration of ART at CD4 counts ≤500 cells/µL for asymptomatic infections; and in the United States guidelines now recommend universal ART, with a stronger recommendation for those with CD4 counts ≤500 cells/µL [5]. With limited health infrastructure and ART resource constraints in many settings, however, strategies are needed to target ART scale-up that maximizes clinical and prevention benefits.

Plasma viral load is the primary predictor of HIV-1 transmission [8], [9] and could be incorporated into ART initiation guidelines to target individuals with an increased risk of transmission [10]. We aimed to compare the potential prevention impact of early ART initiation guidelines (for infected persons with CD4 counts >350 cells/µL) based on high plasma viral load thresholds to guidelines based on CD4 counts ≤500 cells/µL.

## Methods

Data for this analysis were from the Partners in Prevention HSV/HIV Transmission Study, which was a randomized placebo-controlled trial of herpes simplex virus type-2 (HSV-2) suppression with acyclovir among 3381 heterosexual HSV-2/HIV-1 co-infected individuals and their HIV-1 seronegative partners from 7 countries in southern and east Africa (Botswana, Kenya, Rwanda, South Africa, Tanzania, Uganda, and Zambia). At enrollment, all HIV-1 infected participants had CD4 counts ≥250 cells/µL and were not yet on ART. The median CD4 count was 462 cells/µL (interquartile range 348–631) and 68% were women. The primary outcome of the trial was virologically-linked HIV-1 transmission within the partnerships; acyclovir did not decrease the risk of HIV-1 transmission. The design, methods, and primary outcomes have been described previously [11].

For the present analysis, we used cross-sectional data on sexual behavior, CD4 count, and plasma viral load collected at the time of enrollment into the Partners in Prevention HSV/HIV Transmission Study for HIV-1 infected partners with CD4 counts >350 cells/µL. Sexual behavior data were from a standardized questionnaire that asked the number of sex acts with the HIV-1 uninfected partner with and without condoms in the prior month. We used enrollment data only, because our goal was to describe a population presenting to clinical care for consideration of early ART (i.e., at CD4 >350) and thus to minimize the potential impact on sexual behavior reporting as a result of participation in the clinical trial, which included monthly risk-reduction counseling.

We used these cross-sectional data to estimate the number of transmissions that would occur in one year and the number that could be averted with ART in a hypothetical population of 100,000 HIV-1 infected persons in serodiscordant partnerships with CD4 counts and viral load similar to the study participants. To estimate expected transmissions, we first assigned each HIV-1 infected participant per-act infectivity estimates for sex acts with and without condoms, based on our prior analysis of infectivity in the same study population [9]. Notably, in this prior analysis, neither gender nor CD4 count predicted per-act HIV-1 transmission after accounting for reported condom use and plasma viral load. Accordingly, infectivity for the present analysis was estimated solely as a function of reported condom use and viral load. For unprotected acts, this was: λ_1_ = 1– exp{−exp[−6.974+.923*(log_10_ plasma viral load –4)]}, and for acts protected with a condom: λ_0_ = 1–exp{−exp[−8.389+.923*(log_10_ plasma viral load –4)]}. The estimated annual numbers of sex acts with and without condoms were calculated from reported behaviors in the prior month and multiplied by 12. Next, each participant's probability of transmitting to his or her partner within the next year was estimated with the following equation: 1– [(1–λ_0_)^annual #acts with condom^(1–λ_1_) ^annual #acts without condom^]. Because reported condom use was higher in this clinical trial cohort than in previous observational studies of HIV-1 serodiscordant couples, potentially reflecting risk-reduction counseling during the pre-enrollment study screening period, we also calculated each participant's probability of transmitting with reduced condom use (half the amount reported and none). Each participant was assigned a weight of 100,000 divided by the sample size.

Participants were stratified according to plasma viral load (<10,000; 10,000–49,999; ≥50,000 copies/mL) and CD4 count (351–499; ≥500 cells/µL). Several strategies for early ART initiation were considered: CD4 count <500 cells/µL; plasma viral load ≥50,000 or ≥10,000 copies/mL; and universal coverage. The CD4 threshold was based on current international guidelines; viral load thresholds were based on the low risk of transmission with plasma viral loads <10,000 copies/mL [12], and the high risk of transmission at plasma viral loads ≥50,000 copies/mL [2]. Within each early ART scenario, we calculated the number of transmissions potentially averted, assuming a 96% reduction in risk as a result of ART [1]. In a sensitivity analysis, we assumed an 80% reduction in HIV-1 transmission risk, to reflect potential ART prevention benefits outside of a clinical trial [13]. Finally, we estimated the proportion eligible to initiate treatment and the proportion of expected transmissions averted for each potential guideline, and summarized these with a ratio of proportion averted to proportion treated. To visualize the continuous relationship between increasing the proportion initiating treatment and the proportion of infections averted, we further stratified the population by deciles of increasing viral load and by deciles of decreasing CD4 count, and estimated the proportion of infections potentially averted within each decile. We plotted each decile of the population (potentially initiating ART) against the proportion of expected transmissions averted to compare this relationship when ART initiation is based on increasing viral load to the relationship based on decreasing CD4 count.

The clinical trial protocol and informed consent documents were reviewed and approved by human subjects research committees at the University of Washington and all local study site and affiliated institutions. All clinical trial participants provided written informed consent for participating in this research.

## Results

A total of 2484 HIV-1 infected partners had CD4 counts >350 cells/µL, of whom 69% were female. The mean age was 33 years, 42% had CD4 counts <500 cells/µL and 20% had viral loads ≥50,000 copies/mL. Among those with CD4 counts 351–500, the proportion with viral loads ≥50,000 copies/mL was 26%; among those with CD4 counts >500 cells/µL this proportion was 16%. The mean number of reported sex acts per month differed little across CD4 and viral load strata, ranging from 5.2 to 6.6 (Table 1). On average, 77% of sex acts were reported as protected, with negligible differences in reported condom use across strata. About 2% in each strata reported having a sex partner in addition to their known HIV-1 uninfected primary partner. In a hypothetical population of 100,000 HIV-1 infected persons in similar serodiscordant partnerships to our study population, 3,297 HIV-1 transmissions would be expected in one year. Assuming half as much reported condom use, 5,193 transmissions would be expected, and assuming no reported condom use, 6,958.

**Table 1:** Sexual behavior and expected HIV-1 transmissions in one year, stratified by CD4 count and plasma viral load.

					Expected transmissions in 1 year in a population of 100,000**		
			Sex acts per month		Condom use ***		
CD4 cells/µL	Plasma viral load, copies/mL	n (%)	mean (SD)	% protected	Reported	Half	None
350–499	<10,000	455 (18.3)	5.9 (6.6)	77.6	292 (8.9)	466 (9.0)	637 (9.2)
	10,000–49,999	318 (12.8)	6.6 (8.9)	75.5	571 (17.3)	887 (17.1)	1,180 (17.0)
	≥50,000	266 (10.7)	5.2 (5.0)	75.7	771 (23.4)	1,218 (23.5)	1,618 (23.3)
≥500	<10,000	853 (34.3)	6.2 (7.4)	78.5	504 (15.3)	819 (15.8)	1,126 (16.2)
	10,000–49,999	367 (14.8)	5.4 (6.1)	75.1	538 (16.3)	840 (16.2)	1,127 (16.2)
	≥50,000	225 (9.1)	5.3 (6.5)	77.4	621 (18.8)	962 (18.5)	1,271 (18.3)
Totals		3347 (100)			3297 (100)	5193 (100)	6958 (100)

** Data were weighted 100,000/2484 to estimate transmissions in a population of 100,000; The estimated per-act infectivity without a condom was 0.00094 for a person with viral load of 10,000 copies/mL and 0.00178 for a person with viral load of 50,000 copies/mL [9].

*** Reported  =  as reported in the Partners in Prevention HSV/HIV Transmission Study (77% of acts were reported protected), half  =  half the reported frequency of condom use in Partners in Prevention HSV/HIV Transmission Study, none  =  no condom use.

**Figure 1:**
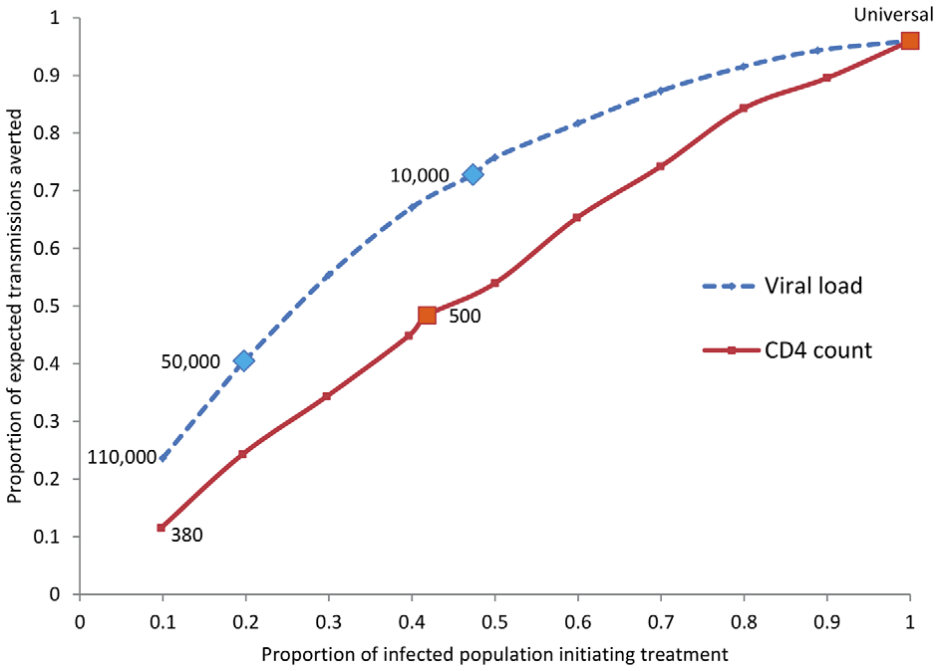
Proportion Initiating Treatment and Expected Transmissions Averted by Decreasing Viral Load and Increasing CD4 Count. The x-axis represents deciles of the population by decreasing viral load or increasing CD4 count; the y-axis represents the proportion of transmissions averted, assuming a 96% reduction in transmission on ART. Overall, the curve based on decreasing viral load begins steeply and later tapers, indicating at the highest viral loads the incremental proportion initiating treatment is small relative to the proportion of infections averted, reflecting the excess of transmissions that occur at high viral loads. The CD4 count curve, by comparison, is linear, suggesting that the prevention benefit associated with increasing the proportion on treatment differs little across CD4 counts in the >350 range.

Initiating treatment at viral load ≥50,000 copies/mL would require treating 19.8% of infected persons with CD4 counts >350 while averting 40.5% of expected transmissions, resulting in a ratio of proportion averted to proportion treated of 2.0 (Table 2). In contrast, initiation at CD4 counts <500 would require treating 41.8%, more than double the amount initiating ART under the high viral load strategy, while averting 48.4% – a ratio of proportion averted to proportion treated of 1.1. If the treatment threshold was based on viral load ≥10,000 copies/mL, this ratio would be 1.5, and for universal treatment the ratio would be 0.96. The proportion initiating treatment relative to the proportion of potential infections averted by decreasing viral load and increasing CD4 count is presented in Figure 1. In a sensitivity analysis assuming a reduction in HIV-1 transmission risk with ART of 80%, the ratio for treatment initiation at viral load ≥50,000 copies/mL was 1.7 (33.8% averted to 19.8% initiating), at viral load ≥10,000 copies/mL was 1.3 (60.7% averted to 47.3% initiating), and at CD4 counts <500 was 0.9 (39.7% averted to 41.8% initiating).

**Table 2:** Comparison of potential guidelines for ART initiation: population size requiring treatment, transmissions averted, and the ratio of % averted to % treated.

	Population eligible for treatment	Expected transmissions averted, n(%)*			Ratio % averted to % initiating		
		Condom use**			Condom use**		
Potential guideline	n (%)	Reported	Half	None	Reported	Half	None
**By CD4 count**							
<500 cells/µL	41,828 (41.8)	1,569 (47.6)	2,469 (47.5)	3,297 (47.4)	1.14	1.14	1.13
**By viral load**							
≥50,000 copies/mL	19,767 (19.8)	1,336 (40.5)	2,093 (40.3)	2,773 (39.9)	2.05	2.04	2.02
≥10,000 copies/mL	47,343 (47.3)	2,401 (72.8)	3,751 (72.2)	4,987 (71.7)	1.54	1.53	1.52
**Universal treatment**	100,000 (100.0)	3165 (96.0)	4985 (96.0)	6679 (96.0)	0.96	0.96	0.96

* Expected transmissions averted shown here is based on a 96% reduction in risk with ART.

** Reported  =  as reported in the Partners in Prevention HSV/HIV Transmission Study (77% of acts were reported protected), half  =  half the reported frequency of condom use in Partners in Prevention HSV/HIV Transmission Study, none  =  no condom use.

## Discussion

Our analysis of different scenarios for ART initiation at earlier than current WHO guidelines indicates that incorporation of plasma viral load, the primary predictor of HIV-1 transmission [8], [9] in ART initiation guidelines could have a greater impact on preventing HIV-1 transmission than ART initiation based on higher CD4 count alone. Recent evidence of the prevention benefits of ART as demonstrated by the HPTN 052 study [1] requires consideration of different scenarios for earlier ART initiation. While scaling up ART access for those who need it their own health is the first priority, targeted ART initiation based on viral load at earlier stages of HIV-1 infection would likely accrue both treatment and prevention benefits.

Others have evaluated the potential impact of initiating treatment based on high viral load in the general population and found strong evidence for an important prevention benefit, consistent with our findings [10]. Our analysis was premised on incorporating high viral load as a staged approach to treatment initiation, after treating all with CD4 counts ≤350, and in direct comparison with raising the CD4 count threshold to 500. Currently, viral load testing is limited in many parts of sub-Saharan Africa and cost widely varies. Efforts are underway to develop lower cost viral load tests [14]. Considering that each transmission, if not averted, could result in greater than 20 years of ART as well as other clinical costs and reduced quality of life, cost-effectiveness analyses are needed of different ART initiation guidelines.

Because we enrolled stable HIV-1-serodiscordant couples who had not transmitted, the cohort could be biased toward HIV-1 infected partners with a lower risk of transmission due to behavioral or biologic factors. However, our population reflects those couples who will present jointly for testing and care, and thus would likely be a population targeted for early ART. In addition, in a community-wide survey in Bostwana, the distribution of viral load among HIV-1 infected persons not on ART with CD4 counts above 350 was similar to that seen in our population [15]. The ratios of infections averted to persons initiating treatment will likely differ somewhat in populations that include HIV-infected single persons, HIV-1 seroconcordant positive couples, or in populations with different distributions of CD4 counts and plasma viral load. However, the relationship between scale up strategies based on increasing viral load to those based on decreasing CD4 count, as shown in the figure and by direct comparison of our estimated ratios, should carry forward to other populations. Because the primary predictor of HIV-1 infectiousness is viral load, the ratio of the proportion of infections averted and the proportion initiating treatment would be even stronger in a population with higher viral loads. Yet in populations in which a large proportion of infected persons with high viral loads are in concordant HIV-1 infected relationships or have otherwise already transmitted, this ratio would be attenuated. In reality, many ART initiation decisions would have to be made without knowledge of partner serostatus, in which case prevention benefits might be less.

A limitation of our analysis is potential inaccuracies in self-reported sexual behavior. The sexual frequency reported in our study is similar to that found in other studies of African heterosexual populations [8], [16]. Importantly, we limited assessment of sexual frequency to that reported at the time of first data collection in our reference population and we estimated expected transmissions with less condom use than what was reported in our clinical trial cohort. Our data, with a large sample size collected over a 2.5 year study enrollment period, captures a broad spectrum of partnerships and risk behavior.

Another limitation is that in using cross-sectional data, our estimates do not incorporate transitions in CD4 count and viral load over time. In reality, individuals would move through CD4 strata gradually, be evaluated for ART periodically (e.g., annually/semi-annually), and become eligible for ART at some point based on CD4 alone. If CD4 counts were to decline relatively faster than plasma viral load increased between re-evaluation visits, our results could be attenuated. While a more complex model could capture these subtleties, because high viral load is such a strong predictor of transmission, guidelines for early ART initiation that include viral load will always avert a higher concentration of new infections than those without viral load.

In conclusion, inclusion of viral load in ART initiation guidelines for those with CD4 >350 cells/µL could be a useful way to increase the prevention benefits of ART by identifying HIV-1 infected persons with the highest risk of HIV-1 transmission. Until resources are available for universal access to ART, it is critical to determine the public health and clinical benefits of ART for persons with higher CD4 counts who are not currently eligible for ART in most resource-constrained settings. A revision of ART guidelines, expanding access to HIV-1 infected persons with CD4 counts >350 cells/µL who have high viral load, could optimize prevention and clinical benefits. Further work is needed to estimate costs, feasibility, and the impact of such guidelines on a population level.
